# Item distribution, internal consistency, and structural validity of the German version of the DEMQOL and DEMQOL–proxy

**DOI:** 10.1186/s12877-018-0930-0

**Published:** 2018-10-19

**Authors:** Christian G. G. Schwab, Martin Nikolaus Dichter, Martin Berwig

**Affiliations:** 1 German Center for Neurodegenerative Diseases (DZNE), Stockumer Straße 12, 58453 Witten, Germany; 2 School of Nursing Science, Witten/Herdecke University, Stockumer Straße 12, 58453 Witten, Germany; 3 Clinic for Cognitive Neurology, Medical Faculty, Leipzig University, Liebigstraße 16, 04103 Leipzig, Germany

**Keywords:** Dementia, Quality of life, Structural validity, Internal consistency

## Abstract

**Background:**

Accurate assessment of health-related quality of life as an endpoint in intervention studies is a major challenge in dementia research. The *DEMQOL* (29 items) and the *proxy* version (32 items), which is partly based on the *DEMQOL*, are internationally used instruments. To date, there is no information on the structural validity, item distribution, or internal consistency for the German language version of these questionnaires.

**Methods:**

This psychometric study is based on a secondary data analysis of a sample of 201 outpatients with a mild form of Alzheimer’s disease (AD) and their informal caregivers. The informal caregivers who were interviewed were involved in the care of the person with AD several times per week. The analysis for the evaluation of the structural validity was performed using Mokken scale analysis. The internal consistency was calculated using the ρ of the Molenaar Sijtsma statistic and Cronbach’s α.

**Results:**

For both versions, four subscales were identified: [A] “positive emotions”, [B] “negative emotions”, [C] “physical and cognitive functioning”, and [D] “daily activities and social relationships”. For both instruments, the internal consistency of all subscales was considered “good” (ρ = 0.71–0.88, α = 0.72–0.87).

**Conclusions:**

The results are a first indication of good construct validity of the instruments used for the German setting. We recommend further investigations of the test-retest reliability and the inter-rater reliability of the proxy instrument.

**Electronic supplementary material:**

The online version of this article (10.1186/s12877-018-0930-0) contains supplementary material, which is available to authorized users.

## Background

According to the World Alzheimer Report, a person was diagnosed with dementia every 3.2 s in 2015. Currently, approximately 46.8 million people worldwide are living with dementia [1]. Dementia is a neurocognitive disorder associated with a significant cognitive decline from a previous level of performance, resulting in a dependency on others to perform activities of daily living [2].

Health-related quality of life (HRQoL) reflects an important desire for persons living with dementia and is therefore used as a general endpoint in many interventional studies. Additionally, HRQoL is increasingly used for assessments of anti-dementia drugs and by the European Medicines Agency to determine the benefits of such drugs [3]. HRQoL in persons living with dementia also is also considered by regulatory authorities and administrative agencies who must judge this parameter based on a resident’s degree of self-sufficiency. Furthermore, HRQoL is used in economic evaluations of persons in all stages of dementia [4–6].

HRQoL is defined by Hays and Reeve as how well a person functions in his/her life and perceives his/her well-being in the physical, mental, and social domains of health [7]. In this definition, functioning refers to the individual’s ability to achieve predefined activities, and well-being refers to individual’s subjective feelings [8, 9]. Based on this and similar definitions of HRQoL, Karimi and Brazier [8] conclude that HRQoL is a particular type of health description; therefore, the World Health Organization (WHO) defines *health* as “a state of complete physical, mental and social well-being, and not merely the absence of disease and infirmity” [10]. This definition indicates that HRQoL measurements reflect health in a wider sense (i.e., well-being and functioning) than solely other clinical outcomes (e.g., 5-year survival rate, rate of restenosis, death, and tumor recurrence).

According to Bakas et al. [11], three models of HRQoL are frequently used: the WHO [12] International Classification of Functioning, Disability and Health (ICF) Model of Functioning and Disability, the HRQoL Model from Wilson and Cleary [9], and, based on this model, the quality of life (QoL) measurement by Ferrans et al. [13].

Smith et al. observed the need to develop a conceptual framework that addressed the differences between the views expressed about HRQoL by people with dementia and their caregivers [14]. The results of the literature analysis and the findings from expert opinions could also be verified by data from interviews of individuals with dementia and their family caregivers [14]. Thus, an empirical justification of the conceptual framework can be assumed. Based on the five domains of the conceptual framework (“health and well-being”, “cognitive functioning”, “daily activities”, “social relationships”, and “self-concept”), they developed two interviewer-administered instruments called Dementia Quality of Life (*DEMQOL*) and its proxy version (*DEMQOL-Proxy*). The authors conducted a pretest factor analysis during the development of the instrument (*DEMQOL n* = 130, *DEMQOL-Proxy n* = 126) that covered the same four dimensions: “positive emotions”, “negative emotions”, “memory”, and “daily activities”. For the *DEMQOL-Proxy*, a two-factor solution, as given by “emotion” and “functioning”, has been suggested by the results of the pretest. The factors of both the self-report and proxy version, however, did not fully support the original conceptual framework [15]. Mulhern and colleagues [4] published a factor analysis with a sample of 644 persons with mild to moderate dementia and 682 proxies. In their study, the subscales “cognition”, “positive emotion” and “negative emotion” could be used on both instruments [4]. However, “social relationships” and “loneliness” were observed only in the *DEMQOL*, while the subscales “daily activities” and “appearance” only occurred in the *DEMQOL-Proxy* [4].

The *DEMQOL* consists of 28 items, while the *DEMQOL-Proxy* includes 31 items on a four-point Likert-type scale with the following responses: a lot, quite slightly, a little, and not at all. The scale includes an additional global QoL item (item 29 resp. 32). Items were scored from 1 to 4, with higher scores indicating better HRQoL. It must be noted that there are five contraindicative items in the *DEMQOL* and the *DEMQOL-Proxy* (4 = a lot, ..., 1 = not at all). The global QoL item also contraindicates the answer options of “very good”, “good”, “fair”, and “poor”. Fifteen items, in addition to the global QoL item, are similar in both versions of the *DEMQOL*; however, there are also items that are not part of the other instrument [15].

The *DEMQOL* can be used in mild to moderate dementia as a self-report form and also for severe dementia in a proxy version (*DEMQOL-Proxy*) across different types of dementia and care arrangements [15]. The utility score (*DEMQOL-U*), which is created from a subset of items from the *DEMQOL*, can also be used for economic assessments [4]. The *DEMQOL* instrument was developed and tested in the UK, which was reported in a Health Technology Assessment (HTA) report [15]. Consequently, it is used more frequently in the UK. While there is a German translation, the results of a linguistic validation have not been reported [16]. No adequate results are available for psychometric testing of the German versions, which are required for both research and applied purposes [17]. To date, the *DEMQOL* has been subjected to at least four more latent variable modeling investigations in two countries since its foundation work [15], two factor analyses [4, 18], bifactor modeling [19], and Rasch modeling [20].

This paper consequently targets the first evaluation of the item distribution, structural validity as a part of the construct validity, and internal consistency of the German version of both the *DEMQOL* and *DEMQOL-Proxy*. For this purpose, a parallel iterative Mokken scale analysis (MSA) is used as a further procedure in addition to the aforementioned methods.

## Methods

The analysis for the present study was performed on a secondary data analysis using anonymous baseline data of a randomized controlled trial called the *Cognitive Rehabilitation and Cognitive Behavioral Treatment for Early Dementia in Alzheimer’s Disease* (CORDIAL) study [21]. To test the structure found in terms of a sensitivity analysis, we used the data of the follow-up surveys after three (T_1_) and after nine months (T_2_). The CORDIAL study was performed to provide clinically meaningful benefits and to evaluate the feasibility, acceptance, efficacy, and usefulness of interventions in cognitive rehabilitation. The study was accomplished as a multicenter randomized controlled trial on persons living with Alzheimer’s disease (AD) and their informal caregivers (as proxy raters). Ethical clearance was granted for the CORDIAL study by the Ethics Commission of the Faculty of Medicine of the Technical University of Munich on 12/10/2009 under the number 2113/08 S. We have refrained from re-auditing as ethical clearance is not required for analyses based on secondary data [22] or for studies using anonymous data.

### Setting and participants

The baseline data of the CORDIAL study were collected from July 2008 to September 2009. The first inclusion criterion required participants to be elderly outpatients with an established ICD-10 diagnosis of AD with mild severity, as defined by a Mini-Mental State Examination (MMSE) score of 21 or above. A differential diagnosis to other forms of dementia was conducted by the recruitment centers. This process was completed to obtain a similar picture of symptoms of the participants, in which memory problems in the early stage are the focus of their everyday problems. Patients were recruited from the ten recruitment centers of the study, including memory clinics and neurological and psychotherapeutic practices throughout Germany.

The need for a designated informal caregiver who was involved in the care of the person living with AD several times a week was the second inclusion criterion for the study. Exclusion criteria were comprised of acute psychiatric or physical disorders, ongoing formal psychotherapy or cognitive training, regular visits to day care facilities, an impending hospital or nursing home admission, a poor command of the German language, alcohol or substance dependence, and participation in another interventional trial. Stable doses of cholinesterase inhibitors, memantine, nootropics, antidepressants, and antipsychotics were permissible as concomitant medications of the person living with AD [21].

### Procedures

The task of the recruitment centers was to inform possible study participants in advance of the study. This process was completed through personal conversations and informational materials. Written informed consent was obtained from both, persons living with AD and their informal caregivers. Independent psychologists, serving as raters, conducted the assessments of the CORDIAL study. The raters received a one-day seminar with case studies for use during the assessments, including the *DEMQOL*-questionnaires. The two interviews with the persons living with AD and the informal caregivers were completed separately. Thus, the informal caregivers were blinded to the answers of the persons living with AD. The monitoring of the study was conducted by an interdisciplinary data monitoring and safety board.

### Measurements

In addition to the previously described *DEMQOL* and *DEMQOL-Proxy*, which are the main topics of interest for our study, further instruments were used. To determine the cognitive ability of the persons living with AD, the German version of the MMSE was used [23]. It is an eleven-question assessment covering five areas of cognitive functioning: orientation, registration, attention and calculation, recall, and language. Each of the 30 tasks is evaluated using a point (range of total scores: 0 to 30). A lower MMSE score indicates a more severe cognitive impairment. To assess impairment of activities of daily living among persons living with AD, the Bayer Activities of Daily Living Scale (B-ADL) by Hindmarch et al. [24] was used. The B-ADL displays a proxy rating assessment for elderly persons with loss of cognitive performance. It is comprised of 25 items rated on a 10-point scale (1 = never, …, 10 = always). The total scores range from 1 to 10, and higher scores indicate higher impairment. To evaluate depressive symptoms, the long form of the Geriatric Depression Scale (GDS) from Yesavage [25] was used. This assessment has 30 dichotomous items (yes or no; directed differently); thus, the total score ranges from 0 to 30 points (higher scores reflect more severe depression). Finally, the Neuropsychiatric Inventory (NPI) by Cummings [26] was used to characterize the neuropsychiatric symptoms and psychopathology of persons living with AD. The NPI covers twelve types of neuropsychiatric disturbances. The frequencies of the symptoms are rated on a 4-point scale multiplied by their severities on a 3-point scale. A higher score (0 to 144) indicates more challenging behavior.

For a description of the informal caregiver, two assessments were used. First, the Beck Depression Inventory (BDI) from Hautzinger et al. [27] is an instrument that assesses the severity of depression. For each of the 21 questions, there are four different answers that are arranged according to their intensity (e.g., item 1: 0 = I do not feel sad; …, 3 = I am so sad or unhappy that I cannot stand it). A higher BDI score (range for total scores: 0 to 63) indicates greater severity of depressive symptoms. Second, the full Zarit Burden Interview (ZBI-22; 22 items) by Zarit et al. [28] measures the subjective burden of informal caregivers, associated with functional/behavioral impairments and the home care situation. The ZBI-22 is rated on a 5-point scale (0 = never, …, 4 = nearly always), and higher scores (range for total scores: 0 to 88) indicates a higher burden.

The socio-demographic data collected for the informal caregiver included age in years and sex, and for the person living with AD, education in years was recorded. In addition, the informal caregiver was asked to explain his/her relationship to the person living with AD.

### Statistical analysis

The descriptions of the participants, missing data, and item distribution were conducted using descriptive statistics. The analysis of the item difficulty was based on the proportion of responses endorsing the best and worst ratings (i.e., ceiling/floor effects). A corresponding effect was assumed conservatively, as long as the mean value of the item was in the lower end (upper 20%) of the respective item range.

For the analysis of the structural validity of the *DEMQOL* and *DEMQOL-Proxy*, as part of the construct validity, we used the MSA. The MSA is a useful tool for researchers who wish to construct unidimensional tests or use questionnaires that comprise multiple binary or polytomous items and enable the examination of reliability without the use of Cronbach’s alpha [29]. The MSA is a method of the non-parametric item response theory originating from assumptions of the unidimensionality of tests or scales, local independency and monotonicity [30]. The method is established in the context of scale development and has been widely used in QoL research [31, 32].

The MSA provides additional information about the relationship between items. As an indicator of the internal correlation of each subscale, the MSA uses Loevinger’s H coefficient (H^S^). According to Sijtsma and Molenaar [33], the following interpretation of H^S^ scores was applied to describe the scale: > 0.5 = “strong”, > 0.4 = “medium”, and >  0.3 = “weak”. The correlation of a single item to the other items of the scale is expressed by the value H_i_. The H_i_ should be non-negative for the Mokken model to hold. Depending on the source, an H_i_-value from 0 to 0.55 is recommended. We have fixed the H_i_-value to the typically used minimum of > 0.3. Items that fall below this level have weak discrimination power and are not useful for this scale. The H_ij_ designated the coefficient of two items.

The criterion (Crit) of the MSA by Molenaar and Sijtsma [34] was used to identify items that partially satisfy the assumptions for monotonous homogeneity or double monotonicity. For each item, this diagnostic value combines the H coefficient, the frequency and size of the violations, and their significance. Every item should have a Crit value of less than 40, and optimally a Crit value of 0. A Crit value of greater than 80 displays a strong indication that an item has violated the assumption for the MSA in this subscale. The critical values were calculated separately for each of the ten imputed records (see below). As a result, individual injuries of double monotonicity should not be systematically increased by a factor of ten.

For the exploratory investigation of the instruments, a method of parallel iteration was used, which consists of two steps. In the first step, cores were determined. The cores are items in an item pool that are strongly correlated with each other (H_ij_) and are less correlated with the other cores when examined as a dyad. This finding means that other items from the pool could improve the H^S^ value in a similar way that a second core could. Otherwise, the weaker second core was returned into the item pool as single items. As a strong correlation for a core, we defined a minimum H_ij_-value of 0.45 as a reference according to Müller-Schneider [35]. Analogous to the procedure in a factor analysis, the number of subscales is thereby predefined. In the second step, an iterative MSA was performed in parallel for each core determined. All items were tested in parallel. Accordingly, all remaining items would be tested against any core, and the item with the highest H_i_-value to a specific core would be chosen. In doing so, the assignment of an item should not lead to a violation of monotonicity (Crit > 40). This procedure was used with regard to content in the case of a possible allocation to two different cores (cross loader). The search procedure stopped when there were no further items that fulfilled the requirements (H_i_ ≥ 0.3 or Crit > 40) or when all the items had been incorporated into a scale. This method of parallel iterative analysis allowed for the identification of smaller subscales with higher H^S^ values, as opposed to the Automated Item Selection Procedure (AISP) for MSA [36].

As a precondition, the MSA assumes only complete cases and integers as values. Therefore, missing values may have to be imputed. In the case of instrument testing, however, the imputation of missing data should be performed with caution. We used a two-way imputation, which is a Bayesian method for estimating missing data in tests and questionnaires [37].

The internal consistency was assessed with the coefficient rho (ρ) of the Molenaar Sijtsma statistic. The ρ coefficient is not as prone to bias as Cronbach’s α and should therefore be preferentially used [38, 39]. For comparison purposes, we also calculated Cronbach’s alpha (α). Values for a ρ or α between 0.70 and 0.95 indicated “good” internal consistency [40]. Finally, we conducted a part-whole-corrected item-total correlation (r_it_) calculation. For this purpose, the coefficient for the item to be examined against the scale without this item was computed. Items with r_it_ coefficients > 0.5 reflected a “high” correlation, and those with r_it_ >  0.3 reflected a “moderate” correlation [41]. An r_it_ correlation of 0.3 and below indicated that the item did not correlate well with the scale as the item may not measuring the same construct as the other variables.

All analysis were performed using R environment for statistical computing version 3.4.1 (30.06.2017) [42]. The following packages were used for the MSA and the imputation, respectively: “mokken” version 2.8.6 [43, 44] and “miceadds” version 2.5–9 [45].

## Results

### Study population

A summary of participant characteristics is shown in Table 1. The total number of participants was 201 dyads (persons living with mild AD and their informal caregivers). Notably, 48% (*n* = 90) of informal caregivers did not specify their relationship to the person with AD (Table 1).

**Table 1:** Participant characteristics

Characteristics	*n* = 201
Persons with dementia	
Age (years)	73.74 (±7.92)
Female	88 (43.8%)
Education level (years)	12.53 (±3.05)
General cognitive ability (MMSE)	25.06 (±2.17)
Functional ability (B-ADL, proxy rating)	3.57 (±1.88) ^a^
Depression (GDS)	8.90 (±5.16)
Challenging behavior (12 types NPI)	9.72 (±9.30)
Informal caregivers (proxy raters)	
Age (years)	64.82 (±13.17)
Female	143 (71.1%)
Spouse of the person with dementia	80 (72.1%) ^b^
Depression (BDI)	8.35 (±6.53) ^a^
Burden (ZBI-22)	19.12 (±12.52)

Data are reported as the mean (±standard deviation) or number (%).*MMSE* Mini-Mental Status Examination, *B-ADL* Bayer Activities of Daily Living Scale;*GDS* Geriatric Depression Scale, *NPI* Neuropsychiatric Inventory;*BDI* Beck Depression Inventory, *ZBI-22* Zarit Burden Interview with 22 items

^a^Missing value in 1 case

^b^Missing values in 90 cases

### Missing value analysis

For the *DEMQOL*, with its 28 items and with 201 participants, there were seven missing responses (0.1%), while in the proxy version of the *DEMQOL* (31 items), there were eleven missing responses (0.2%; Table 2). The missing values corresponded to seven cases of one missing item (3.6%) in the *DEMQOL*, as well as nine cases in which one item (3.2%) and one case in which two items (6.5%) were missing in the *DEMQOL-Proxy*. Ten complete datasets with two-way imputed integers for missing data were generated for the MSA.

**Table 2:** Item distribution on the German version of the *DEMQOL* and *DEMQOL-Proxy* (No. of original item order; *n* = 201)

*DEMQOL*									*DEMQOL-Proxy*								
No.	Item	Response options				Descriptive			No.	Item	Response options				Descriptive		
		a lot	quite a bit	a little	not at all	missing	mean (±SD)				a lot	quite a bit	a little	not at all	missing	mean (±SD)	
Feelings In the last week, have you felt …									Feelings In the last week, would you say that *(your relative)* has felt …								
1.	…cheerful? ^a^	85	90	24	2	0	3.28	(±0.71)	1.	…cheerful? ^a^	67	91	41	2	0	3.11	(±0.75)
2.	…worried or anxious?	22	65	60	54	0	2.73	(±0.98)	2.	…worried or anxious?	31	90	50	29	1	2.39	(±0.92)
3.	…that you are enjoying life? ^a^	110	61	27	3	0	3.38	(±0.77)									
4.	…frustrated?	14	43	75	69	0	2.99	(±0.92)	3.	…frustrated?	15	73	72	41	0	2.69	(±0.88)
5.	…confident? ^a^	82	77	34	8	0	3.16	(±0.85)									
6.	…full of energy? ^a^	93	75	26	6	1	3.28	(±0.80)	4.	…full of energy? ^a^	42	77	62	20	0	2.70	(±0.91)
7.	…sad?	17	59	64	61	0	2.84	(±0.96)	5.	…sad?	14	86	68	33	0	2.60	(±0.84)
									6.	…content? ^a^	104	72	22	2	1	3.39	(±0.72)
8.	…lonely?	12	24	51	114	0	3.33	(±0.91)									
9.	…distressed?	6	23	62	110	0	3.37	(±0.80)	7.	…distressed?	27	94	51	29	0	2.41	(±0.90)
10.	…lively? ^a^	73	74	38	16	0	3.01	(±0.94)	8.	…lively? ^a^	55	76	52	18	0	2.84	(±0.93)
11.	…irritable?	19	50	74	57	1	2.85	(±0.95)	9.	…irritable?	30	75	63	32	1	2.49	(±0.93)
12.	…fed-up?	4	40	54	103	0	3.27	(±0.85)	10.	…fed-up?	12	69	64	56	0	2.82	(±0.91)
									11.	…that he/she has things to look forward to? ^a^	80	92	26	3	0	3.24	(±0.73)
13.	…that there are things that youwanted to do but couldn’t?	8	42	68	82	1	3.12	(±0.88)									
Memory In the last week, how worried have you been about …									Memory In the last week, how worried would you say *(your relative)* has been about …								
									12.	…his/her memory in general?	89	73	26	13	0	1.82	(±0.89)
									13.	…forgetting things that happened a long time ago?	28	58	73	42	0	2.64	(±0.96)
14.	…forgetting things that happened recently?	59	79	35	28	0	2.16	(±1.00)	14.	…forgetting things that happened recently?	85	66	33	15	2	1.89	(±0.94)
15.	…forgetting who people are?	26	44	42	88	1	2.96	(±1.09)									
									15.	…forgetting people’s names?	52	79	45	24	1	2.21	(±0.96)
									16.	…forgetting where he/she is?	12	24	61	104	0	3.28	(±0.90)
16.	…forgetting what day it is?	27	51	50	73	0	2.84	(±1.07)	17.	…forgetting what day it is?	36	56	50	59	0	2.66	(±1.08)
17.	…your thoughts being muddled?	32	55	56	58	0	2.70	(±1.05)	18.	…his/her thoughts being muddled?	30	68	54	48	1	2.60	(±1.01)
18.	…difficulty making decisions?	11	51	54	85	0	3.06	(±0.95)	19.	…difficulty making decisions?	29	54	66	51	1	2.70	(±1.01)
19.	…poor concentration?	63	65	43	29	1	2.19	(±1.04)									
24.	…making yourself understood? ^b^	14	31	57	99	0	3.20	(±0.94)	20.	…making him/herself understood?	12	48	56	85	0	3.06	(±0.95)
Everyday life In the last week, how worried have you been about …									Everyday life In the last week, how worried would you say *(your relative)* has been about …								
20.	…not having enough company?	4	17	51	129	0	3.52 ^d^	(±0.74)	28.	…not having enough company?	5	25	49	121	1	3.43^d^	(±0.81)
21.	…how you get on with people close to you?	8	19	47	126	1	3.46 ^d^	(±0.83)									
22.	…getting the affection that you want?	4	17	42	137	1	3.56 ^d^	(±0.73)									
23.	…people not listening to you?	9	25	50	117	0	3.37	(±0.87)									
25.	…getting help when you need it?	4	23	42	132	0	3.50 ^d^	(±0.78)									
26.	…getting to the toilet in time?	12	20	20	149	0	3.52 ^d^	(±0.90)									
27.	…how you feel in yourself?	14	29	45	113	0	3.28	(±0.95)									
28.	…your health overall?	28	67	49	57	0	2.67	(±1.04)									
									21.	…keeping him−/herself clean(e.g. washing and bathing)?	2	7	23	169	0	3.79^d^	(±0.55)
									22.	…keeping him−/herself looking nice?	6	18	41	136	0	3.53^d^	(±0.78)
									23.	…getting what he/she wants from the shops?	3	25	49	124	0	3.46^d^	(±0.77)
									24.	…using money to pay for things?	12	23	38	128	0	3.40^d^	(±0.91)
									25.	…looking after his/her finances?	14	38	37	112	0	3.23	(±0.99)
									26.	…things taking longer than they used to?	33	61	48	58	1	2.66	(±1.07)
									27.	…getting in touch with people?	1	31	61	108	0	3.37	(±0.76)
									29.	…not being able to help other people?	3	22	58	118	0	3.45^d^	(±0.75)
									30.	…not playing a useful part in things?	12	41	41	107	0	3.21	(±0.97)
									31.	…his/her physical health?	37	73	42	48	1	2.51	(±1.05)
Frequencies of the response options in total, n		**860**	**1316**	**1340**	**2105**	**7**			Frequencies of the response options in total, n		**978**	**1785**	**1522**	**1935**	**11**		
Frequencies of the response options in total, %		**15.3**	**23.4**	**23.8**	**37.5**	**0.1**			Frequencies of the response options in total, %		**15.7**	**28.7**	**24.5**	**31.1**	**0.2**		
No.	Item	Response options				Descriptive			No.	Item	Response options				Descriptive		
		very good	good	fair	poor	missing	mean (±SD)				very good	good	fair	poor	missing	mean (±SD)	
OverallThinking about all of these things in the last week, how would you rate …									OverallThinking about all of these things in the last week, how would you say *(your relative)* would rate …								
29.	…your quality of life overall?^c^	23	139	37	2	0	2.91	(±0.58)	32.	…his/her quality of life overall? ^a^	11	125	58	7	0	2.70	(±0.63)
Frequencies of the response options, %		**11.4**	**69.2**	**18.4**	**1.0**	**0.0**			Frequencies of the response options, %		**5.5**	**62.2**	**28.9**	**3.5**	**0.0**		

^a^Contraindicative items

^b^The item corresponds to both versions, however in the original questionnaire it is part as number 24 of “Everyday life”

^c^Contraindicative items

^d^Ceiling effect, the mean is in the last 20% of the scale (≥ 3.4)

### Item distribution

Five items (20–22, 25, 26) for the *DEMQOL* and six items (21–24, 28, 29) for the *DEMQOL-Proxy* showed a ceiling effect (Table 2). The 15 identical items of the *DEMQOL* and *DEMQOL-Proxy* demonstrated that proxy ratings are typically lower than the corresponding self-ratings (Table 2).

### Structural validity

Four cores were found for the *DEMQOL*: items 21/24, 7/9, 17/19, and 6/10 (all H_ij_ ≥ 0.49). In addition, for the *DEMQOL-Proxy*, four cores were found, consisting of items 4/8, 12/14, 5/7, and 23/24 (all H_ij_ ≥ 0.65). The results of the iterative MSA are shown in Table 3. We found four equal subscales for both instruments, i.e., [A] “positive emotions”, [B] “negative emotions”, [C] “physical and cognitive functioning”, and [D] “daily activities and social relationships” (Table 3).

**Table 3:** Scale analysis and internal consistency of the *DEMQOL* and *DEMQOL-Proxy* (*n* = 201)

*DEMQOL*		ρ	α	H^S^			*DEMQOL-Proxy*		ρ	α	H^S^		
A.	Positive emotions	**0.71**	**0.72**	**0.38**	H_i_	r_it_	A.	Positive emotions	**0.88**	**0.86**	**0.66**	H_i_	r_it_
10.	…lively?				0.41	0.52	8.	…lively?				0.71	0.78
6.	…full of energy?				0.39	0.50	4.	…full of energy?				0.65	0.69
1.	…cheerful?				0.37	0.47	11.	…that he/she has things to look forward to?				0.66	0.70
3.	…that you are enjoying life?				0.37	0.46	1.	…cheerful?				0.66	0.69
5.	…confident?				0.34	0.43	6.	…content?				0.58	0.59
B.	Negative emotions	**0.80**	**0.80**	**0.45**	H_i_	r_it_	B.	Negative emotions	**0.79**	**0.81**	**0.46**	H_i_	r_it_
7.	…sad?				0.52	0.66	7.	…distressed?				0.51	0.65
9.	…distressed?				0.51	0.63	5.	…sad?				0.51	0.65
8.	…lonely?				0.41	0.50	3.	…frustrated?				0.53	0.67
4.	…frustrated?				0.47	0.58	2.	…worried or anxious?				0.43	0.53
12.	…fed-up?				0.41	0.49	10.	…fed-up?				0.46	0.55
2.	…worried or anxious?				0.40	0.48	9.	…irritable?				0.31	0.37
C.	Physical and cognitive functioning	**0.83**	**0.82**	**0.42**	H_i_	r_it_	C.	Physical and cognitive functioning	**0.88**	**0.87**	**0.50**	H_i_	r_it_
19.	…poor concentration?				0.50	0.66	12.	…his/her memory in general?				0.50	0.59
17.	…your thoughts being muddled?				0.47	0.64	14.	…forgetting things that happened recently?				0.50	0.60
14.	…forgetting things that happened recently?				0.42	0.53	15.	…forgetting people’s names?				0.51	0.62
27.	…how you feel in yourself?				0.40	0.49	18.	…his/her thoughts being muddled?				0.57	0.75
18.	…difficulty making decisions?				0.42	0.55	19.	…difficulty making decisions?				0.52	0.66
28.	…your health overall?				0.40	0.53	17.	…forgetting what day it is?				0.49	0.63
16.	…forgetting what day it is?				0.37	0.49	16.	…forgetting where he/she is?				0.46	0.53
13.	…that there are things that you wanted to do but couldn’t?				0.33	0.43	20.	…making him/herself understood?				0.46	0.54
							13.	…forgetting things that happened a long time ago?				0.45	0.57
D.	Daily activities and social relationships	**0.84**	**0.83**	**0.46**	H_i_	r_it_	D.	Daily activities and social relationships	**0.86**	**0.86**	**0.42**	H_i_	r_it_
21.	…how you get on with people close to you? ^a^				0.49	0.65	23.	…getting what he/she wants from the shops? ^a^				0.48	0.67
24.	…making yourself understood?				0.50	0.62	24.	…using money to pay for things? ^a^				0.43	0.59
23.	…people not listening to you?				0.52	0.68	21.	…keeping him−/herself clean (e.g. washing and bathing)? ^a^				0.45	0.50
22.	…getting the affection that you want? ^a^				0.49	0.63	27.	…getting in touch with people?				0.49	0.67
25.	…getting help when you need it? ^a^				0.47	0.62	22.	…keeping him−/herself looking nice? ^a^				0.45	0.59
20.	…not having enough company? ^a^				0.39	0.49	30.	…not playing a useful part in things?				0.42	0.58
26.	…getting to the toilet in time? ^a^				0.34	0.40	29.	…not being able to help other people? ^a^				0.42	0.58
							26.	…things taking longer than they used to?				0.40	0.51
							31.	…his/her physical health?				0.38	0.45
							28.	…not having enough company? ^a^				0.38	0.52
							25.	…looking after his/her finances?				0.36	0.48

The criterion (Crit) by Molenaar, Sijtsma, and Boer used to check the monotonicity assumption is for all items in every of the ten imputed datasets = 0

ρ = rho (Molenaar Sijtsma statistic); α = Cronbach’s alpha (> 0.7 and < 0.95 = good); r_it_ = part-whole-corrected item-total correlation (> 0.50 = high, > 0.30 = moderate);

H^S^ = Loevinger’s H coefficient of scalability of the scale (> 0.50 = strong, > 0.40 = medium, > 0.30 = weak); H_i_ = Loevinger’s H coefficient for an item and the remaining items of the scale

^a^ Item with ceiling effect (see Table 2)

For the *DEMQOL*, the H^S^ coefficient showed a “medium” correlation for three subscales (H = 0.42–0.46) in comparison to the subscale [A], which presented only a “weak” (H = 0.37) correlation. The correlations of the *DEMQOL-Proxy* subscales are “strong” ([A] H^S^ = 0.66) and “medium” subscales ([C] H^S^ = 0.50; [B] H^S^ = 0.46; [D] H^S^ = 0.42). Subscale [A] differed between both instruments, with only a “weak” H^S^ in the *DEMQOL* but a “strong” H^S^ in the *DEMQOL-Proxy* version. The MSA used all items of the proxy version, while in the *DEMQOL*, two items (11: “irritable”; 15: “forgetting who people are”) could not be assigned. With regard to content, item 11 could have matched to subscale [B], while item 15 would have matched to subscale [C]. However, both showed a H_i_ of < 0.3. Item 24 from the *DEMQOL*, “making yourself understood”, and the corresponding item 20 of the *DEMQOL-Proxy* version, “making him/herself understood”, are in different subscales ([C] vs. [D]). Apart from this finding and the missing item 11 in the *DEMQOL* version, the remaining 13 of the 15 identical items of the two instruments have been assigned to the same subscales. In the *DEMQOL*, the item 18 (“difficulty making decisions”) presented as a cross loader, which could have assigned to subscale [C] with H_i_ = 0.45 as well as to subscale [D] with H_i_ = 0.39. For a better fit with respect to content and the conceptual framework of Smith et al. [15], we decided to move item 18 into subscale [C]. The assumption of monotonicity for the MSA has been achieved for each of the ten imputed samples for all items (Crit = 0). All items with a ceiling effect could be found in subscale [D].

Sensitivity analyses were carried out to determine the stability of the identified subscales using the data from the CORDIAL study follow-up surveys, which can be found in Additional file 1: Table A1 for time T_1_ after three months and in Additional file 2: Table A2 for the nine-month survey (T_2_) in the supplemental material of this article. With the exception of item 26 in the *DEMQOL* version at time T_2_, the results were confirmed. The H^S^ values in the *DEMQOL* version are comparable to the baseline values with “medium” correlations for subscale [B] to [D] and to a “weak” correlation for subscale [A] (Additional file 1: Table A1). At T_2_, the assessment on subscale [A] improved to a “medium”, whereas subscale [D] marginally worsened. The subscales [B] and [C] of the *DEMQOL-Proxy* improved to “strong” for both timepoints. The same result was found for subscale [D] at T_2_. Therefore, at T_2_, all subscales of the *DEMQOL-Proxy* are “strong” (Additional file 2: Table A2).

### Internal consistency

The subscales revealed a ρ of 0.71–0.84 (α = 0.72–0.83) for the *DEMQOL* and a ρ of 0.79–0.88 (α = 0.81–0.87) for the *DEMQOL-Proxy*. According to the ρ- and α-values, all subscales were considered “good” (Table 3). The internal consistency was also “good” for T_1_ and T_2_ for all subscales, with ρ- and α-values > 0.72 (Additional file 1: Table A1 and Additional file 2: Table A2). The r_it_ revealed only one item (item 9 from the *DEMQOL-Proxy*, “irritable”) that was below 0.4, which was not scalable at all in the *DEMQOL* version. All other items had a “high” (*n* = 41) or “moderate” (*n* = 15) r_it_-values of 0.4 or more.

## Discussion

The evaluation of the German versions of the *DEMQOL* and *DEMQOL-Proxy* revealed the following major findings. First, the MSA for the *DEMQOL* and the *DEMQOL-Proxy* revealed that four subscales ([A] “positive emotions”, [B] “negative emotions”, [C] “physical and cognitive functioning”, and [D] “daily activities and social relationships”) were found with stable H^S^ values ≥0.38. Second, the internal consistency showed consistently “good” values. Third, the item description showed ceiling effects, as exhibited by 18% of the items on the *DEMQOL* and 19% of the items on the *DEMQOL-Proxy*. Furthermore, the results of the MSA for the German versions of the *DEMQOL* and *DEMQOL-Proxy* are largely comparable to findings from previous studies on the HRQoL of persons living with dementia [4, 15], especially to the conceptual framework of Smith and colleagues [14] as well as to their explorative factor analysis of the pretest during the development of the instrument. As such, our results can be considered to be first indications of the structural validity of the *DEMQOL* and the *DEMQOL-Proxy*.

In our data evaluation, the domain “health and well-being” of the conceptual framework is represented by subscales [A], “positive emotions”, and [B], “negative emotions”, except items 27 (“how you feel in yourself”) and 28 (“your health overall”), which have been loaded onto subscale [C], “physical and cognitive functioning”. Therefore, this subscale has been complemented by the word “physical”. For the *DEMQOL-Proxy*, only item 31 (“his/her physical health”) from the framework was loaded onto subscale [D], “daily activities and social relationships”. Subscale [C] characterizes the domain “cognitive functioning” of the conceptual framework in our data evaluation. Herein, 100% conformity for the *DEMQOL-Proxy* could be shown. However, item 24 of the *DEMQOL* (“making yourself understood”) loaded onto subscale [D]. The domains “daily activities” and “social relationships” of the framework were combined into the fourth subscale of our study, which was designated as subscale [D], “daily activities and social relationships”. However, item 13 of the *DEMQOL* (“that there are things that you wanted to do but couldn’t”) was an exception, as it was loaded to the subscale [C], “physical and cognitive functioning”. This loading may be due to the translation of the word “inability” into German. Furthermore, item 30 of the *DEMQOL-Proxy* (“not playing a useful part in things”) was added to subscale [D]. This was the only item that could be assigned to the “self-concept” domain of the conceptual framework.

Compared to the findings of Mulhern and colleagues, we also found subscales for “positive emotions” and “negative emotions” in our study. In contrast, in our assessment, subscale [C], “physical and cognitive functioning”, is related to what has been termed “cognition” in the HTA report by Mulhern and colleagues [4]. The subscales “social relationships” in the *DEMQOL* and “daily activities” in the *DEMQOL-Proxy* were referred to the common subscale [D], “daily activities and social relationships”, for both instruments within our data evaluation. Taken together, 71% of the *DEMQOL* items and 65% of the *DEMQOL-Proxy* items coincided with the results of Mulhern et al. [4]; that is, 20 items that are equally distributed in both instruments.

Furthermore, the subscales “positive emotions” (both instruments) and “negative emotions” (*DEMQOL-Proxy* only) are completely identical, if the cross loaders of the *DEMQOL-Proxy* in the Mulhern study are not removed. Subscale [B] of the *DEMQOL*, “negative emotions”, exhibits a difference, as given by the loading of items 8 and 9, while item 11 is not loaded. In contrast, the subscale “cognition” shows high consistencies with the *DEMQOL* and *DEMQOL-Proxy*. Differences in the *DEMQOL*, however, exist only in the additional loading of the items 13, 27 and 28, while item 15 was not loaded. On the *DEMQOL-Proxy*, item 26 was not loaded, while all other items were identical. Item 20 of *DEMQOL* was additionally loaded within subscale [D], “daily activities and social relationships”, which was named “social relationships” by Mulhern et al. [4]. Only three items of the *DEMQOL-Proxy* could be seen in the data set of Mulhern et al. [4], while our data evaluation further loaded the items 21, 22, and 26–31.

In summary, our data demonstrated the determined subscales to be highly consistent with the conceptual framework of Smith et al. [15] and that these subscales further exhibit similarities to those of Mulhern et al. [4]. The sensitivity analysis at times T_1_ and T_2_ showed a stable result for assignment of the items to the subscales (Additional file 1: Table A1 and Additional file 2: Table A2). In contrast, the designation of subscale [D] was identical in the *DEMQOL* and *DEMQOL-Proxy*, while this finding differed according to Mulhern et al. [4] and was therefore presented as “social relationships” in the *DEMQOL* and “daily activities” in the *DEMQOL-Proxy*. However, if both subscales were taken together, they represent a similar construct as our subscale [D], “daily activities and social relationships”.

In accordance with the HRQoL definition from Hays and Reeve [7] provided in the background, the subscales we found in our study cover the aspects of “how well a person functions in his/her life” (physical and cognitive functioning [C]) and his or her “perceived well-being in physical (daily activities), mental (positive and negative emotions [A-B]), and social domains (social relationships [D]) of health”.

### Limitations

The presented data of this psychometric study used datasets from the CORDIAL study by Kurz et al. [21]. Thus, since additional data could not be obtained, no statements on the inter-rater reliability of the *DEMQOL-Proxy* can be made to estimate the quality of the underlying data. Similarly, it was no longer possible to influence the number of study participants. According to a study by Straat et al. [46], a sample of more than 250 respondents should be given if the quality of the answers is high. In the present study, however, only the data of 201 persons could be used, reflecting a limitation of the results. Furthermore, the CORDIAL study included only persons with a mild severity of AD. The mild form might explain the ceiling effects of subscale [D]. Thus, the generalizability of the results is limited due to the absence of other forms of dementia.

## Conclusions

In this psychometric study using the German versions of the instruments *DEMQOL* and *DEMQOL-Proxy*, four equal subscales were found in both instruments, demonstrating “good” internal consistency. The subscales reflect the conceptual framework of the instrument developers to a high degree. Thus, the results can be considered a first indication of the construct validity of the two German versions. In our opinion, *DEMQOL* subscale scores are more explanatory than a total score because HRQoL is a multidimensional concept and respective domain scores may help clarify treatment impacts. Moreover, our internal consistency results reflect the homogeneity of the subscales. However, Chua et al. [19] used bifactor models for direct comparisons between total and subscale scores and showed that the latter scores had poor reliability and should not be used. Such direct comparisons were not performed in our study due to differences in modeling decisions (parallel iterative MSA rather than bifactor modeling). The merits of MSA and bifactor modeling for clarifying multidimensionality are debatable [47, 48]. Therefore, more empirical data are needed before definitive recommendations can be made.

However, we recommend further investigations prior to or integrated in future studies using the German version of the *DEMQOL* and *DEMQOL-Proxy*. In particular, we advise the integration of the evaluation of test-retest reliability (both *DEMQOL* versions) and inter-rater reliability (*DEMQOL-Proxy*) as part of the instrument application in future studies. Continued research should also be carried out on structural validity using various latent variable models. In addition, further investigation of data from persons living with moderate dementia, or with severe dementia for the proxy version, should be performed according to the ceiling effects we found in our study. For the proxy version, it would also be important to conduct a study with professional nurses to make statements on the use of the instrument in German nursing homes. Similarly, an investigation should be conducted to analyze against external criteria for other proportions of the construct validity.

## Additional files

Additional file 1: **Table A1.** Scale analysis and internal consistency of the *DEMQOL* (*n* = 183) and *DEMQOL-Proxy* (*n* = 179) using the data of T_1_, three months after the baseline assessment. (PDF 240 kb)

Additional file 2: **Table A2.** Scale analysis and internal consistency of the *DEMQOL* (*n* = 166) and *DEMQOL-Proxy* (*n* = 163) using the data of T_2_, nine months after the baseline assessment. (PDF 316 kb)

## Abbreviations

AD: Alzheimer’s disease
B-ADL: Bayer Activities of Daily Living Scale
BDI: Beck Depression Inventory
CORDIAL: Cognitive Rehabilitation and Cognitive Behavioral Treatment for Early Dementia in Alzheimer’s Disease (study)
Crit: Criterion by Molenaar, Sijtsma, and Boer
DEMQOL: Dementia Quality of Life
GDS: Geriatric Depression Scale
H_i_: Loevinger’s H coefficient for an item and the remaining items of the scale
HRQoL: Health-related quality of life
H^S^: Loevinger’s H coefficient of scalability of a scale
HTA: Health Technology Assessment
ICF: International Classification of Functioning, Disability and Health
MMSE: Mini-Mental Status Examination
MSA: Mokken scale analysis
NPI: Neuropsychiatric Inventory
QoL: Quality of life
r_it_: Part-whole-corrected item-total correlation
SD: Standard deviation
WHO: World Health Organization
ZBI-22: Zarit Burden Interview with 22 items
α: Cronbach’s alpha
ρ: rho (Molenaar Sijtsma statistic)
