# Supplementary material for: Item distribution, internal consistency, and structural validity of the German version of the DEMQOL and DEMQOL–proxy
Source: BMC Geriatr. 2018 Oct 19;18:247. doi: 10.1186/s12877-018-0930-0 (PMC6194625; doi:10.1186/s12877-018-0930-0)
Supplement: Supplementary file 2 — Table A2. Scale analysis and internal consistency of the DEMQOL (n = 166) and DEMQOL-Proxy (n = 163) using the data of T2, nine months after the baseline assessment. (PDF 316 kb) [file 12877_2018_930_MOESM2_ESM.pdf]

Appendix

Table A2: Scale analysis and internal consistency of the DEMQOL (n = 166) and DEMQOL-Proxy (n = 163) using the data of T2, nine months after the baseline assessment.

DEMQOL	ρ	α	H^S	H_i	r_{it}
**A. Positive emotions**	**0.78**	**0.77**	**0.45**	**H_i**	**r_{it}**
10. …lively?				0.51	0.62
6. …full of energy?				0.43	0.52
1. …cheerful?				0.44	0.53
3. …that you are enjoying life?				0.43	0.50
5. …confident?				0.45	0.56
**B. Negative emotions**	**0.81**	**0.80**	**0.45**	**H_i**	**r_{it}**
7. …sad?				0.53	0.68
9. …distressed?				0.45	0.55
8. …lonely?				0.35	0.41
4. …frustrated?				0.48	0.61
12. …fed-up?				0.41	0.49
2. …worried or anxious?				0.48	0.60
**C. Physical and cognitive functioning**	**0.87**	**0.86**	**0.49**	**H_i**	**r_{it}**
19. …poor concentration?				0.54	0.68
17. …your thoughts being muddled?				0.57	0.72
14. …forgetting things that happened recently?				0.52	0.63
27. …how you feel in yourself?				0.53	0.65
18. …difficulty making decisions?				0.52	0.67
28. …your health overall?				0.45	0.56
16. …forgetting what day it is?				0.38	0.47
13. …that there are things that you wanted to do but couldn't?				0.44	0.54
**D. Daily activities and social relationships**	**0.80**	**0.80**	**0.40**	**H_i**	**r_{it}**
21. …how you get on with people close to you? [a]				0.43	0.60
24. …making yourself understood?				0.41	0.53
23. …people not listening to you?				0.46	0.65
22. …getting the affection that you want? [a]				0.40	0.54
25. …getting help when you need it? [a]				0.48	0.68
20. …not having enough company? [a]				0.29	0.38
26. ...getting to the toilet in time? [a]				*0.28*	*0.35*

DEMQOL-Proxy	ρ	α	H^S	H_i	r_{it}
**A. Positive emotions**	**0.87**	**0.85**	**0.62**	**H_i**	**r_{it}**
8. …lively?				0.70	0.79
4. …full of energy?				0.61	0.67
11. …that he/she has things to look forward to?				0.58	0.62
1. …cheerful?				0.65	0.70
6. …content?				0.54	0.56
**B. Negative emotions**	**0.85**	**0.85**	**0.53**	**H_i**	**r_{it}**
7. …distressed?				0.56	0.68
5. …sad?				0.54	0.64
3. …frustrated?				0.58	0.69
2. …worried or anxious?				0.53	0.62
10. …fed-up?				0.57	0.69
9. …irritable?				0.42	0.49
**C. Physical and cognitive functioning**	**0.91**	**0.90**	**0.51**	**H_i**	**r_{it}**
12. …his/her memory in general?				0.60	0.70
14. …forgetting things that happened recently?				0.56	0.65
15. …forgetting people's names?				0.52	0.60
18. …his/her thoughts being muddled?				0.57	0.70
19. …difficulty making decisions?				0.54	0.66
17. …forgetting what day it is?				0.58	0.71
16. …forgetting where he/she is?				0.58	0.67
20. …making him/herself understood?				0.55	0.64
13. …forgetting things that happened a long time ago?				0.54	0.65
**D. Daily activities and social relationships**	**0.91**	**0.90**	**0.51**	**H_i**	**r_{it}**
23. …getting what he/she wants from the shops? [a]				0.54	0.71
24. …using money to pay for things? [a]				0.54	0.70
21. …keeping him-/herself clean (e.g. washing and bathing)? [a]				0.56	0.69
27. …getting in touch with people?				0.54	0.70
22. …keeping him-/herself looking nice? [a]				0.56	0.70
30. …not playing a useful part in things?				0.50	0.64
29. …not being able to help other people? [a]				0.51	0.66
26. …things taking longer than they used to?				0.47	0.57
31. …his/her physical health?				0.43	0.48
28. …not having enough company? [a]				0.53	0.69
25. …looking after his/her finances?				0.41	0.52

The criterion (Crit) by Molenaar, Sijtsma, and Boer used to check the monotonicity assumption is for all items in every of the ten imputed datasets = 0, only item 20 of *DEMQOL* has a crit value on average of 56.
ρ = rho (Molenaar Sijtsma statistic); α = Cronbach's alpha (> 0.7 and < 0.95 = good); r_{it} = part-whole-corrected item-total correlation (> 0.50 = high, > 0.30 = moderate);
H^S = Loevinger's H coefficient of scalability of the scale (> 0.50 = strong, > 0.40 = medium, > 0.30 = weak); H_i = Loevinger's H coefficient for an item and the remaining items of the scale.
[a] Item with ceiling effect, the mean is in the last 20 percent of the scale (≥ 3.4).
